# Role of Pelvic Ultrasound in Predicting the Response to Neoadjuvant Chemotherapy in Locally Advanced Cervical Cancer

**DOI:** 10.3390/diagnostics15040463

**Published:** 2025-02-14

**Authors:** Giorgia Perniola, Giulia Paoni Saccone, Noemi Tonti, Federica Tanzi, Innocenza Palaia, Violante Di Donato, Federica Tomao, Ludovico Muzii, Giorgio Bogani, Ilaria Cuccu, Enrico Ciminello, Francesco Antonio Battaglia, Giusi Santangelo

**Affiliations:** 1 Department of Maternal and Child Health and Urological Sciences, Policlinico Umberto I, Sapienza University of Rome, 00161 Rome, Italy; giorgia.perniola@uniroma1.it (G.P.); giulia.paonisaccone@uniroma1.it (G.P.S.); federica.tanzi@uniroma1.it (F.T.); innocenza.palaia@uniroma1.it (I.P.); violante.didonato@uniroma1.it (V.D.D.); federica.tomao@uniroma1.it (F.T.); ludovico.muzii@uniroma1.it (L.M.); ilaria.cuccu@uniroma1.it (I.C.); giusi.santangelo@uniroma1.it (G.S.); 2 Gynecological Oncology Unit, Fondazione IRCCS Istituto Nazionale dei Tumori, 20133 Milan, Italy; giorgio.bogani@istitutotumori.mi.it; 3 Istituto Superiore di Sanità, 00161 Rome, Italy; enrico.ciminello@iss.it; 4 Department of Gynecology and Obstetrics, Ospedale Santa Maria Goretti, 04100 Latina, Italy; franbatt@alice.it

**Keywords:** cervical cancer, NACT, US in cervical cancer, RIUA, predictive role of ultrasound, radical surgery, presurgical staging

## Abstract

**Background/Objectives**: The optimal treatment for locally advanced cervical cancer (LACC) is debated. The proposed treatments are concomitant chemoradiotherapy plus brachytherapy (cCTRT-B) or neoadjuvant chemotherapy (NACT) followed by radical surgery (RS). The prediction NACT response is crucial for identifying responder patients who may benefit from subsequent radical surgery. The aim of this study was to find ultrasound characteristics to predict the response to NACT in patients with LACC. **Methods**: Consecutive patients with diagnoses of LACC were prospectively enrolled. According to FIGO staging criteria, all IB2-IIIC patients underwent three cycles of platinum-based NACT followed by radical surgery. Patients were evaluated by pelvic ultrasound one week before NACT (T0) and three weeks after the last cycle of chemotherapy (T1). The parameters analysed were volume of the lesion, tumor/uterus volume ratio, parametrial infiltration, color score, resistance (RIUA) and pulsatility (PIUA) indices of uterine arteries (UA). **Results**: From July 2019 to April 2023, 40 patients were enrolled. A significant decrease in tumor volume (*p* < 0.01) and a reduced parametrial infiltration after NACT were observed (*p* < 0.01). The results of the unadjusted and adjusted logistic models showed that age and RIUA positively affect the estimated probability of treatment response (*p* < 0.01). According to the univariate and multivariate model, RIUA greater than 0.72 ensures 87% sensitivity and 70% specificity with 82.5% accuracy in predicting tumor reduction. **Conclusions**: Patients over 54 with a RIUA above 0.72 are more likely to respond to NACT. Pelvic ultrasound proved to be a useful tool for predicting NACT response in LACC patients.

## 1. Introduction

According to the 2020 Global Cancer Statistics, cervical cancer (CC) is still the second most diagnosed cancer and the fourth cause of cancer death in women, with an incidence of 604,000 new cases and 342,000 deaths worldwide [1]. The International Federation of Gynecology and Obstetrics (FIGO) classification system (revised in 2018) is widely used for staging CC [2]. It categorizes the disease based on tumor size, local extension, lymph node involvement, and distant metastasis, ranging from Stage I (early localized disease) to Stage IV (advanced metastatic disease). This classification not only guides the choice of treatment but also provides prognostic insights. According to international guidelines [3], treatment strategies for cervical cancer are stage dependent. Early-stage disease (FIGO Stages IA–IIA1) is often treated surgically with curative intent. Palliative care is reserved for metastatic (Stage IVB) or recurrent disease when curative treatment is no longer feasible, focusing on symptom relief and improving quality of life. While the standard treatment for locally advanced cervical cancer (LACC), FIGO stage IB3-IVA, is concomitant chemoradiation plus brachytherapy (cCTRT-B), neoadjuvant chemotherapy (NACT) followed by radical surgery (RS) has been extensively used in specialized centers and seems to have almost comparable results in terms of overall survival (OS) and progression-free survival (PFS), as demonstrated in two randomized trials [4,5]. Indeed, findings from previous studies indicate that CC is a chemosensitive tumor [6]. For instance, platinum-based NACT is effective in shrinking tumor volume and eradicating micrometastatic disease, facilitating radical operability while minimizing impact on pelvic structures and the vagina [7,8]. Furthermore, data in the literature regarding long-term toxicity showed a higher rate of vaginal, rectal and bladder post-treatment sequelae in patients who underwent CTRT compared to NACT plus RS [5]. On the other hand, the response rate of platinum-based NACT is approximately 60% [9]. These data suggest that NACT may be ineffective in some instances, potentially delaying definitive treatment and increasing the risk of tumor progression. Moreover, in patients who do not respond to chemotherapy, radiation therapy also appears to be generally ineffective [10,11]. Therefore, the prediction of NACT response is crucial. Even if magnetic resonance imaging (MRI) is considered the gold standard for the staging of CC, particularly in IB1-IIIC FIGO stage tumors [12,13], transvaginal ultrasound (TV-US) or transrectal ultrasound (TR-US) have shown promising results in evaluating the local extension of the disease [14,15]. Our previous study [16] showed how US can be considered a valid tool to assess the NACT response. The aim of this study was to identify US features that could predict the response to NACT in LACC patients.

## 2. Materials and Methods

This prospective study enrolled consecutive patients who were referred to the Department of Oncological Gynecology, Policlinico Umberto I of Rome, with a histological diagnosis of uterine cervical cancer. The research was approved by the local ethics commission. All patients signed an informed consent. All clinical, histopathological, and imaging data were correctly stored in an appropriate database. Patients were enrolled according to the following inclusion criteria: female aged ≥ 18 years and <75 years; histological diagnosis of cervical squamous cell carcinoma or cervical adenocarcinoma; clinically documented LACC (FIGO IB2-IIIC stage); patients with cervical carcinoma not treated before; performance status ECOG score 0–2 [17]. Exclusion criteria: rare histology (neuroendocrine, sarcoma); concomitant tumors; concomitant severe disease (chronic heart failure NYHA class III–IV; chronic kidney disease with GFR < 30 mL/min; cirrhosis with Child-Pugh class C); distant metastases; performance status ECOG score > 2 [17]. Patients and the public were not involved in the design, conduct, reporting or dissemination plans of our research. All patients underwent a clinical examination performed by an experienced oncological gynecologist to evaluate the clinical stage of the disease. This procedure included a visit under anesthesia with bimanual palpation, both vaginally and rectally, and the performance of cervical biopsies. In case of suspected involvement of the rectum or bladder, a cystoscopy and a proctoscopy were performed. All patients underwent a CT scan to exclude the presence of distant metastases. Patients with a confirmed histological and radiological diagnosis of LACC were submitted to 3 cycles of platinum-based NACT (Cisplatin 75 mg/m^2^ or Carboplatin AUC = 5 plus paclitaxel 175 mg/m^2^) every 21 days (or a weekly regimen in selected cases, mainly for frail patients). All patients underwent 2D/3D TV-US and MRI within one week before NACT (T0) and three weeks after the last cycle of chemotherapy (T1). 2D/3D TR-US was performed in patients who had never had sexual intercourse and in patients with severe bleeding or vaginal/cervical pain. The responses to NACT were evaluated following the RECIST criteria (version 1.1) for solid tumors [18]. Patients who were considered eligible for surgery underwent radical surgery within 40 days after the last NACT cycle. Chemotherapy-related severe toxicity that required a suspension of the treatment and a progressive disease were the main reasons for not undergoing radical surgery per protocol. This included radical hysterectomy, bilateral salpingo-oophorectomy and systematic pelvic lymphadenectomy. Patients of reproductive age with squamous cell carcinoma or HPV-associated adenocarcinoma were counselled about the possibility of preserving the ovaries [3]. US findings were compared with histopathological results to test the accuracy of 2D/3D TV-US in assessing response to NACT.

### 2.1. Ultrasound Technique

The ultrasound exam was performed by an oncological gynecologist with more than ten years of experience in ultrasound gynecological tumors. A GE Voluson 730 (GE HealthCare, Little Chalfont, Buckinghamshire, UK), equipped with a 7.5 MHz transvaginal probe of 7.5 MHz and a 3.5–5 MHz transabdominal convex probe of 3.5–5 MHz, was adopted. Firstly, a transabdominal ultrasound examination was performed with a full bladder. The investigator systematically evaluated the uterus, ovaries, kidneys, and the presence of any hydroureteronephrosis. In patients with a BMI < 30, transabdominal ultrasound was useful to evaluate the presence of bulky lymph nodes. Then, an ultrasound examination was performed with an empty bladder by a transvaginal probe, systematically evaluating the tumor lesion—which looks like a hypo-isoechoic lesion with irregular walls—the uterus and ovaries, bladder, ureters, parameters and rectosigmoid area. The 3D analysis was carried out in a similar way to the conventional 2D analysis. Modifications of the tumor volume, the vascular and infiltration pattern and the uterine artery velocimetry indices were recorded at every US examination.

### 2.2. Measure and Reference Standard

The US features analyzed: (I) the three main diameters of the uterus to calculate the uterine volume; (II) the three main diameters of the lesion to calculate the tumor volume; (III) the tumor/uterus volume ratio; (IV) the extension of the tumor to the first, second, or third area of the vaginal canal; (V) the infiltration of vesicovaginal and rectovaginal septum (evaluating the anterior and posterior sonographic “sliding sign”); (VI) the parametrial infiltration, classified into 3 grades according to tumor diffusion, as grade 1 (i.e., mild involvement of the parametrium), grade 2 (i.e., moderate involvement of the parametrium) and grade 3 (i.e., complete involvement of the parametrium), as previously described [15]. The tumor vascularization was qualitatively assessed using a Color Doppler (with PRF 0.3–0.6 kHz, WF 50 Hz, high gain). A subjective semiquantitative evaluation of intratumor flow (Color score) was performed, assigning score 1 to no identified flow; score 2 to minimal flow; score 3 to moderate flow and score 4 to high degree of vascularization. Bilateral velocimetry of the uterine artery was performed and wave morphology, the resistance index of the uterine arteries (RIUA), and the pulsatility index (PIUA) were analyzed. Other features analyzed were: (I) the histotype, (squamous or adenosquamous); (II) the histological grading, (G1, G2, or G3); (III) the stage, from IB2 to IIIC, according to the 2019 International Federation of Gynecology and Obstetrics (TNM) classification.

### 2.3. Statistical Analysis

The sample size was defined to ensure at least 95% significance with at least 90% precision for the estimation of the mean in single arm studies with quantitative variable as outcome [19], while considering the percentage reduction of the tumor between T0 and T1 as the primary outcome and assuming a 30% SD. Descriptive analysis was conducted on paired samples at T0 and T1: continuous variables are reported in terms of mean (SD), while categorical variables are reported in terms of frequency (percentage); differences between the two observation occasions were investigated using the Wilcoxon test and the McNemar test, respectively. The percentage reduction of the tumor was considered a target variable: the impact of other features on tumor reduction was investigated by a linear model, unadjusted and adjusted, to account for possible confounders and features interaction. A patient responding or non-responding to treatment was determined according to the RECIST criteria (version 1.1) for solid tumors [18]. Comparison between responders and non-responders to treatment was performed using the Mann–Whitney test and χ2 test was used to assess the significance of differences for continuous and categorical variables, respectively. The impact of patient characteristics on responding/not responding was investigated using logistic regression. Model selection was carried out by stepwise approach and evaluated using adjusted R squared for linear models and Akaike Information Criterion, adjusting for the number of parameters and avoiding overfitting for logistic regressions. Finally, the performance of the model was evaluated in terms of the area under the curve (AUC). An interaction analysis was carried out after scaling covariates to check for possible interactions between predictors after model selection. A sensitivity/specificity study was implemented, and operating characteristics were used to select optimal models and thresholds for predictors. Statistical analysis was performed using the software, R, version 4.2.3 (15 March 2023 ucrt)—“Shortstop Beagle”.

## 3. Results

**Figure 1:**
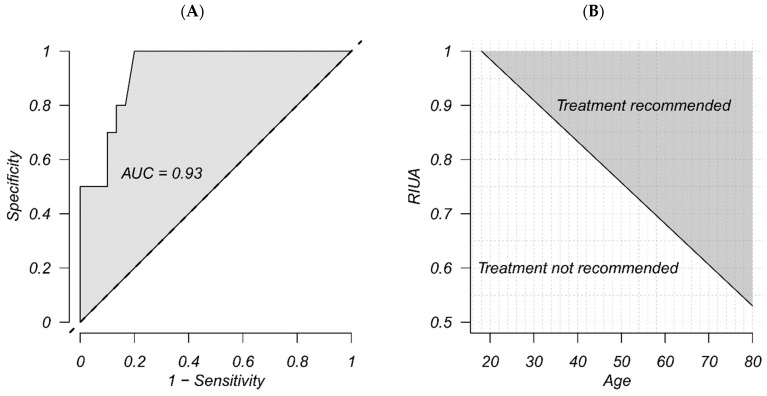
(**A**): ROC curve for best multivariate logistic model. Responders as outcome. (**B**): Optimal threshold for RIUA and age combined, derived from the best multivariate logistic model.

From July 2019 to April 2023, a total of 40 consecutive patients affected by LACC were enrolled in this study. Table 1 showed patients’ characteristics at T0 and T1. Briefly, 34 (85%) patients had squamous cell carcinoma while 6 (15%) had adenocarcinoma. Most patients 24 (60%) had a IIIC FIGO stage; no patients with IIIA or IIIB FIGO stage were detected at the time of the diagnosis because all patients with suspicious lymph nodes were classified as FIGO stage IIIC, regardless of whether they had involvement of the lower third of the vagina (FIGO IIIA) or showed tumor extension to the pelvic wall with hydronephrosis (FIGO IIIB). A G3 was recorded in 47.5% (n = 19) women. The tumor volume decreased by 17.9 cm^3^ in size, on average, after treatment (*p* < 0.01). After chemotherapy, a statistically significant reduction in parametria infiltration was observed, both in cases of severe and moderate infiltration (*p* < 0.01), confirming the well-documented effectiveness of NACT [15,20]. The exploration of possible predictors of tumor reduction among the characteristics of patients at T0 is reported in Table 2. Only the age of the patients (*p* = 0.01) and RIUA (*p* < 0.01) showed a significant correlation with tumor size reduction, both with a positive effect, even when adjusting for possible confounders. The percentage of responders to NACT according to RECIST criteria [16] was equal to 75% (n = 30). Differences in clinical characteristics in T0 between responders and non-responders are reported in Table 3: the only significant differences were found in age (53.77 vs. 40.5 years) and RIUA (0.81 vs. 0.72), both significantly higher in responders (*p* < 0.01). Age and RIUA positively affect the estimated probability of a patient responding to NACT. The model considering both combined features (Table 4) showed the best performance (AUC = 0.93) and significance for both coefficients (*p* < 0.01). The interaction analysis performed with such covariates did not detect a significant interaction between age and RIUA in the selected model (*p* = 0.65), while coefficient estimates for age and RIUA were significant (*p* = 0.03) and close to the significance threshold (*p* = 0.07), respectively (Table 5). This also confirmed the absence of collinearity between predictors in the model, as correlation between age and RIUA was equal to 0.03. A sensitivity/specificity study was performed to identify the optimal threshold to predict responders using RIUA alone or both RIUA and age as predictors. According to the univariate model, treating patients with RIUA greater than 0.72 ensures 87% sensitivity and 70% specificity with 82.5% accuracy in predicting responsiveness. Including age as a predictor, while sensitivity remained at 87%, specificity and accuracy increased to 80% and 85%, respectively. The optimal threshold for combined RIUA and age is shown in Figure 1.

Tables and Figures legend: RIUA resistance index uterine artery; PIUA pulsatility index uterine artery.

## 4. Discussion

The evidence established in this study showed that older patients with a higher RIUA (>0.72) are more likely to respond to NACT. Including age as a clinical parameter increases specificity, improving patient selection for effective treatment and reducing unwanted side effects. Our research group has already conducted a prospective study that evaluated the role of the US in predicting the early response to NACT analyzing tumor volume and lesion vascularization index (VI) reduction [16]. In patients classified as responders, a significant reduction in tumor size and VI was already observed after two cycles of chemotherapy. In line with our results, the potential of 3D power Doppler ultrasound in predicting response to chemotherapy was also investigated in another study that recruited 61 patients diagnosed with LACC eligible for NACT followed by radical surgery [21]. The analysis identified the flow index (FI) as the only useful marker for predicting both clinical and histological responses to chemotherapy in patients with LACC, which is higher in responders than in non-responders. The physio–pathological explanation for this result is that the FI value is directly related to the degree of oxygenation of the tumor and, as demonstrated by other studies in the literature, tumors with a high degree of hypoxia are resistant to standard therapies [22]. Data regarding intra-tumor vascularization are strongly present in the literature [23]; conversely there are few data on extra-tumor vascular assessment in patients with LACC who underwent NACT, as focused on this study. Indeed, we evaluated the predictive role of the 2D–US flow indices of the uterine arteries in this setting. Our result, identifying an increase in RIUA as a feature that affects tumor reduction, is in line with a previous work investigating RI values in uterine arteries conducted by Greco et al. [24]. This study compared the RI values of the uterine arteries and tumor vessels in patients with CC and in healthy patients. RI values were found to decrease significantly in patients with CC. Furthermore, patients who responded positively to chemotherapy showed a post-CHT increase in RI values in uterine arteries. Post-CHT change in RI values is clinically related to a change in tumor vascularization, associated with a reduction in the size of the lesion, in patients considered responders. An explanation of this phenomenon can be found in the consolidated studies on neoangiogenesis in the oncological field: the neoplastic mass needs for its growth an abundant vascularization, which is guaranteed from the neovessels, which are characterized by the absence of a smooth muscle layer and the presence of numerous arteriovenous shunts. Both these anatomical peculiarities are related to an increase in intratumor blood flow velocity and low resistance [25]. In addition, tumor cells secrete many vasoactive molecules and growth factors that promote neoangiogenesis. A key role in this area is played by vascular endothelial growth factor (VEGF) that is synthesized directly by neoplastic cells in response to a hypoxic stimulus. Hypoxia induces cell death by apoptosis; in contrast, neoplastic cells activate resistance mechanisms, such as the production of VEGF, essential to ensure their survival. VEGF advances neoangiogenesis by stimulating endothelial proliferation, increasing vascular permeability and promoting a vasodilatory effect through the release of nitric oxide [26]. This vasodilator effect could explain the subsequent alterations in flow in the uterine arteries. Furthermore, as demonstrated by Testa et al., tumors with larger diameter (≥4 cm) showed a reduction in the RI values of intratumoral vessels compared to smaller tumors [27]. Therefore, based on these data, it is possible to assume that higher RI values of uterine arteries correspond to less aggressive clinical behavior of the tumor, and a more likely response to NACT. In addition, as already demonstrated in the previous literature [28], the evidence found in our study shows that age positively affects the estimated probability to respond to treatment. The impact of age on prognosis in CC is controversial. An explanation of this evidence could be found in the role of cellular senescence: some hallmarks of ageing can mediate both an oncogenic and an oncosuppressive action. There are several age-associated mechanisms that usually limit cellular fitness and proliferation and plasticity, such as inhibition of autophagy, cell senescence, stem cell exhaustion and telomere attrition, which provides an oncosuppressive effect. The induction of senescence and the elimination of senescent cells have been proposed as possible anti-cancer mechanisms that could explain our findings [29]. Huang et al. also suggested that bulky tumors in younger patients may be biologically more aggressive and therefore their outcome is less affected by NACT compared to older patients [30].

The main strength of our study is that US evaluation has been prospectively performed before and during treatment, blinded to pathological reports.

The main limitations of the study are the monocentric design, the small sample size and ultrasound reproducibility issues due to device variability. Future studies based on a wider sample size should aim to establish standard, reproducible ultrasonographic protocols.

Data reported in this study can contribute to the treatment of local advanced cervical cancer, as well as facilitating continued refinement of future guidelines, systematic reviews and meta-analyses. This study’s data support using skilled gynecologist-performed ultrasound as a valid, accurate and cost-effective tool to assess NACT response in locally advanced cervical cancer. Including age in assessment algorithms may improve patient selection for NACT + RS.

## 5. Conclusions

Cervical cancer remains the second most diagnosed tumor in women, especially in low-income countries, often presenting at a locally advanced stage. This study, based on a single institution’s experience with LACC patients, reinforces the role of uterine flow indices and introduces RI as a predictive marker for NACT response. Additionally, it highlights age as a potential prognostic factor. Multi-institutional studies with larger populations are needed to confirm these findings, standardize US parameters, and correlate them with clinical and histopathological data.

## Figures and Tables

**Table 1:** Patients features comparison: before (T0) and after (T1) NACT.

	T0 (SD or %)	T1 (SD or %)	*p*-Value
Age	50.4 (12.1)	-	-
Uterus volume (cm^3^)	54.1 (17.76)	-	-
Histotype			
Squamous	34 (85%)	-	-
Adenosquamous	6 (15%)	-	-
Stage			
IB2-IIB	16 (40%)	-	-
IIIC	24 (60%)	-	-
Grading			
G2	21 (52.5%)	-	-
G3	19 (47.5%)	-	-
Tumor size (cm^3^)	28.9 (19.5)	11 (11.6)	<0.01 *
Parametrium infiltration			<0.01 *
No infiltration	9 (22.5%)	24 (60%)	
Moderate infiltration	18 (45%)	11 (27.5%)	
Severe infiltration	13 (32.5%)	5 (12.5%)	
Color Scale	3 (0.83)	2 (0.79)	<0.01 *
Sliding sign			
No	21 (52.5%)	15 (37.5%)	<0.01 *
Anterior	0 (0%)	0 (0%)	
Posterior	10 (25%)	5 (12.5%)	
Both	9 (22.5%)	20 (50%)	
PIUA	1.88 (0.79)	1.72 (0.61)	0.38
RIUA	0.78 (0.09)	0.79 (0.1)	0.30
Tumor/uterus volume ratio	0.49 (0.22)	0.18 (0.17)	<0.01 *

The asterisk (*) placed next to the variables was used to highlight variables that were statistically significant or close to statistical significance.

**Table 2:** Linear models results. Tumor reduction as outcome.

	Coefficient Estimate (SD)	Confidence Interval	*p*-Value
Age	0.009 (0.003)	[0.002, 0.02]	0.01 *
Uterus volume (cm^3^)	−0.0005 (0.003)	[−0.006, 0.005]	0.85
Histotype			
Squamous	−0.09 (0.13)	[−0.35, 0.16]	0.46
Adenosquamous	baseline		-
Stage			
IIA-IIB	baseline		-
IIIC	−0.14 (0.09)	[−0.32, 0.05]	0.14
Grading			
G2	baseline		-
G3	−0.06 (0.09)	[−0.25, 0.12]	0.48
Tumor size (cm^3^)	0.0002 (0.002)	[−0.004, 0.005]	0.91
Parametrium infiltration			
No infiltration	baseline		
Moderate infiltration	0.09 (0.12)	[−0.14, 0.33]	0.42
Severe infiltration	0.11 (0.12)	[−0.15, 0.36]	0.40
Color Scale	−0.01 (0.05)	[−0.12. 0.1]	0.82
Sliding sign			
No	−0.10 (0.11)	[−0.33, 0.13]	0.40
Anterior	-	-	-
Posterior	−0.13 (0.13)	[−0.4, 0.14]	0.34
Both	baseline		-
Iliac lymph nodes (cm)	0.01 (0.06)	[−0.12, 0.14]	0.84
PIUA	0.06 (0.06)	[−0.05, 0.18]	0.27
RIUA	1.55 (0.44)	[0.65, 2.45]	<0.01 *
Tumor/uterus volume ratio	0.06 (0.21)	[−0.36, 0.47]	0.78

The asterisk (*) placed next to the variables was used to highlight variables that were statistically significant or close to statistical significance.

**Table 3:** Comparison between responders and non-responders characteristics before NACT.

	Responders	Non-Responders	*p*-Value
n	30 (75%)	10 (25%)	
Age	53.77 (11.24)	40.5 (8.75)	<0.01 *
Uterus volume (cm^3^)	55.5 (18.44)	49.9 (15.68)	0.5
Histotype			
Squamous	24 (80%)	10 (100%)	0.31
Adenosquamous	6 (20%)	0 (0%)	
Stage			
IIA-IIB	13 (43.33%)	3 (30%)	0.71
IIIC	17 (56.67%)	7 (70%)	
Grading			
G2	15 (50%)	6 (60%)	0.85
G3	15 (50%)	4 (40%)	
Tumor size (cm^3^)	30.10 (19.73)	25.40 (19.41)	0.13
Parametrium infiltration			0.59
No infiltration	6 (20%)	3 (30%)	
Moderate infiltration	13 (43.33%)	5 (50%)	
Severe infiltration	11 (36.67%)	2 (20%)	
Color Scale	3.03 (0.67)	2.8 (1.23)	0.84
Sliding sign			
No	16 (53.34%)	5 (50%)	0.91
Anterior	-	-	
Posterior	7 (23.33%)	3 (30%)	
Both	7 (23.33%)	2 (20%)	
Iliac lymph nodes (cm)	0.34 (0.66)	0.48 (0.89)	0.7
PIUA	1.94 (0.86)	1.71 (0.51)	0.55
RIUA	0.81 (0.08)	0.72 (0.07)	<0.01 *
Tumor/uterus volume ratio	0.51 (0.22)	0.46 (0.23)	0.64

The asterisk (*) placed next to the variables was used to highlight variables that were statistically significant or close to statistical significance.

**Table 4:** Best multivariate logistic model. Responders as outcome.

	Coefficient Estimate (SD)	Confidence Interval	*p*-Value
Age	0.14 (0.05)	[0.05, 0.27]	<0.01 *
RIUA	18.44 (7.76)	[5.83, 37.29]	0.02 *

The asterisk (*) placed next to the variables was used to highlight variables that were statistically significant or close to statistical significance.

**Table 5:** Interaction in the best multivariate logistic model. Responders as outcome.

	Coefficient Estimate (SD)	Confidence Interval	*p*-Value
Age	1.92 (0.87)	[0.5, 4.1]	0.03 *
RIUA	2.03 (1.13)	[0.29, 4.9]	0.07
Interaction	0.47 (1.03)	[-1.49, 2.8]	0.65

The asterisk (*) placed next to the variables was used to highlight variables that were statistically significant or close to statistical significance.

## Data Availability

The data sets generated for this study are available on request from the corresponding author.

## Abbreviations

The following abbreviations are used in this manuscript:

LACC	locally advanced cervical cancer
cCTRT-B	concomitant-chemoradiotherapy-brachytherapy
NACT	neoadjuvant chemotherapy
RS	radical surgery
RIUA	resistance index uterine artery
PIUA	pulsatility index uterine artery
CC	cervical cancer
OS	overall survival
PFS	progression free survival
CHT	chemotherapy
